# Does the immediate reimbursement of medical insurance reduce the socioeconomic inequality in health among the floating population? Evidence from China

**DOI:** 10.1186/s12939-023-01913-7

**Published:** 2023-05-17

**Authors:** Wen He

**Affiliations:** School of Public Administration, Hunan University, Lushan Road (S), Yuelu District, Changsha, 410082 China

**Keywords:** Socioeconomic status, Health inequality, Immediate reimbursement, Medical insurance, Floating population

## Abstract

**Background:**

Enhancing health intervention for floating populations has become an essential aspect of public health around the world. China launched a policy reform aimed at implementing immediate reimbursement for trans-provincial inpatient treatments. The objective of this study was to investigate the effects of this policy change on socioeconomic inequality in health among the floating population.

**Methods:**

This study used two waves of individual-level data from the China Migrants Dynamic Survey (CMDS) collected in 2017 and 2018 as well as administrative hospital data at the city level. The sample included 122061 individuals and 262 cities. Under a quasi-experimental research design, we built up the framework to employ the generalized and multi-period difference-in-differences estimation strategy. We used the number of qualified hospitals that could provide immediate reimbursement to represent the degree and intensity of the implementation of this policy change. We also calculated the Wagstaff Index (WI) to measure socioeconomic inequality in health.

**Results:**

This policy change and income level had a negative joint impact on the health status of floating population (odds ratio = 0.955, *P* < 0.01), that is, the lower the income, the better the number of qualified hospitals' effect on health improvement. Furthermore, as the number of qualified tertiary hospitals increased, the health inequality would decrease significantly on average at the city level (*P* < 0.05). In addition, inpatient utilization as well as total expenditure and reimbursement significantly improved after the policy change, and the magnitude of increase was greater in the relatively lower-income group (*P* < 0.01). Finally, only inpatient spending could obtain immediate reimbursement in the early stage, thus, compared with primary care, these impacts were greater in tertiary care.

**Conclusions:**

Our study revealed that after the implementation of immediate reimbursement, the floating population could obtain greater and more timely reimbursement, which significantly increased its inpatient utilization, promoted health, and reduced the health inequality caused by socioeconomic factors. These results suggest that a more accessible and friendly medical insurance scheme should be promoted for this group.

## Background

With the acceleration of regional economic integration, the improvement of transportation convenience, and acceleration of population ageing, many people choose to leave their hometowns for places with more developed economies or better climate conditions to improve the quality and happiness of their lives [1, 2]. Undeniably, migrants inject impetus into the development of local areas and obtain marked development opportunities for themselves, but they still cannot enjoy equal social support and security as local residents. That is, most migrants remain marginalized [3, 4]. This group is usually engaged in the construction, transportation, and delivery industries, which primarily require strength and time, and these jobs are mostly informal and temporary, resulting in an unstable and low income level [5, 6]. The pressure to survive, broken social relationships, changed lifestyle and living environment poses consistent health risks for this population, and the losses of social health security worsen these conditions and widen the income disparities, deteriorate the competitive environment and intensify social contradictions [7, 8]. This phenomenon is extremely common in China, whose floating population is one of the largest migrant populations in the world. According to the data released by the National Bureau of Statistics [9], the floating population reached 375.8 million by 2020, 33.2% of which was trans-provincial. These migrants usually have a lower status than local permanent residents and mainly come from rural and inland areas [10, 11], which is associated with distinct health disadvantages [12, 13].

The pursuit of equality is a key objective of healthcare systems, and worldwide consensus holds that everyone equally owns the right to health and equal health need ought to be treated equally regardless of income, identity, race or any other factors [14]. However, individuals actually have different socioeconomic conditions, with high earners receiving better health education and enjoying better medical accessibility and so on, which bring better health outcome [15]. Thus, socioeconomic inequality in health indeed exists no matter in developed countries or developing countries [16–19]. To promote public health and alleviate health inequality, it is not only necessary for individuals to develop a positive and upward lifestyle, but also for government to carry out relevant overall planning. Medical insurance has been extensively linked to numerous health benefit, and this health benefit is documented to be more effective for low-income groups, thereby helping reduce socioeconomic inequality in health [20, 21]. Accordingly more and more countries are devoting to establishing medical insurance scheme and expanding its coverage.

China has basically achieved universal medical insurance coverage, which makes remarkable contributions to alleviating medical burdens and promoting health of citizens [22]. Nowadays, this huge system consists of two public medical insurance schemes: Urban Employee Basic Medical Insurance (UEBMI) and Urban and Rural Resident Basic Medical Insurance (URRBMI). UEBMI is mandatory and designed for individuals with formal employment, whereas URRBMI is voluntary and specified for nonworking populations, such as children, students, or individuals with informal employment. In particular, this medical insurance system executes localized administration strategies, and the risks are pooled at the municipal or county level [23]. That is, when the enrolees utilize medical services out of the place where they enrol in the scheme, the reimbursement will not be immediate, and the cost-sharing ratios will increase too. Therefore, the insured must pay the full cost for each treatment and receive reimbursement later. The floating population lives outside their place of household registration. Due to their occupations, they usually do not have the economic strength to enrol in the local UEBMI scheme, whose contribution is much higher than that of the URRBMI [23]. In addition, they face invisible policy constrains to enrol in local URRBMI, which is specified for local permanent residents [24]. That is, most individuals in this population generally have to enrol in the medical scheme in the place of household registration, resulting in sharp increases in the time cost and medical burden. Thus, the distinct health disparity between local residents and the floating population is not surprising. Furthermore, marked socioeconomic inequality in health exists among the floating population because high-income groups always have more opportunities and methods to utilize social networks and resources to promote health, and this phenomenon may be ignored [15]. The insufficient security will further heighten this inequality.

To promote social equality and improve the health conditions of the floating population, the Chinese government has declared that it will implement immediate reimbursement for trans-provincial inpatient treatment [25]. This policy took effect on January 1, 2017. Subsequently, when enrolees utilized inpatient services outside the place of insurance registration, they were also able to obtain immediate reimbursement and the reimbursement ratios will improve too. However, only enrolees that visit hospitals that have access to the National Online Reimbursement System (hereinafter referred to as qualified hospitals) can enjoy this convenience. By September 2022, 61 thousand hospitals were qualified, comprising almost 6% of all hospitals. The fund paid for immediate reimbursement reached 7.9 billion yuan and the actual reimbursement ratio was up to 60.6% [26].

Increasing the convenience of reimbursement is not unique to China; some countries and regions such as The European Union and Ghana advanced similar reforms, and these have been found to be associated with significant improvements in health promotion [27, 28]. However, the distribution of medical resources is uneven in practice, and high-income groups have better access to medical care and thus likely receive more reimbursement from medical insurance [29]. Health equality may even deteriorate under these circumstances. Although related studies of the health benefits of medical insurance have produced highly informative results, studies of the impact of the reimbursement method remain limited. After the implementation of immediate reimbursement, the trans-provincial floating population only needs to pay the coinsurance rate for inpatient treatments after exceeding the deductible, which improves the convenience and accessibility of reimbursement. Thus, this policy change should be linked with positive health effects. However, in China, except for the uneven distribution of medical resources, there are still some limitations on reimbursement accessibility, such as definite categories [30]. Therefore, the present study aimed to empirically explore the effect of this policy, which could provide evidence for the further improvement of this policy in China and inform other countries to help solve problems due to medical insecurity.

By using two waves of data from the China Migrants Dynamic Survey collected in 2017 and 2018 as well as the administrative data of hospitals and employing the generalized and multi-period difference-in-differences estimation strategy, we obtained the following results. First, there was significant pro-rich health care inequality among floating population. Second, after the immediate reimbursement implemented, the convenience of reimbursement increased, which significantly promoted its inpatient utilization and reduced its medical burdens. This impact was greater in the relatively low-income group. Thus, as the number of hospitals that could provide immediate reimbursement increased, the health status of the floating population significantly improved. In addition, the lower the income, the better the number of qualified hospitals' effect on health improvement; that is, it helped reduce socioeconomic inequality in health. Finally, these effects were more effective in tertiary care than other hospitals because this policy change only covered inpatient services currently.

This article enriches the literature on the relationship between medical insurance and health status of floating population. Many studies have found that migrants are in bad health conditions for the inaccessibility of health care [27, 28], while patients are sensitive to cost, health insurance is an effective method to promote the improvement of their medical care utilization and health status [31–33]. In addition, patients are more likely to utilize medical services when cost sharing is low and reimbursement is timely [34–36]. The results of our paper are consistent with these findings. In particular, our paper enriches the related literature in China by examining the impacts of reimbursement method change. Most related literature has generally focused on the effects of medical insurance coverage [37, 38], but examining the impact of policy change, such as patient cost-sharing and reimbursement methods, has become increasingly essential because universal medical insurance coverage has basically been achieved in China [22].

In addition, our study also contributes to the related research literature on how to alleviate healthcare inequality. Many studies have explored the ability of medical insurance policy to help reduce health disparities [20, 21], and some of them have investigated the impacts of urban–rural resident medical insurance integration in China and demonstrated that integration has significantly impacts the medical utilization of rural residents and positively affects the promotion of urban and rural equality in terms of healthcare utilization [39–41]. Based on these studies, our study targeted the floating population, and to the best of our knowledge, this study is one of the first to investigate whether and how the reimbursement method impacts health inequality.

Finally, this work adds to a growing body of literature regarding the health effects of primary care. This body of literature has shown that the utilization of primary care is positively associated with primary and secondary prevention, chronic health condition management, reduced hospital utilization, and congestion [42–44]. Several studies further empirically examined methods to encourage the use of primary care, such as conditional cash-transfer programs [45] and changes in patient cost-sharing [46]. However, the findings we obtained contrast those of previous research, which can bring some enlightenment for the optimization of its functional localization.

## Methods

### Data source

The individual-level data were derived from two waves of the China Migrants Dynamic Survey (CMDS) collected in 2017 and 2018. This survey covers 31 provinces in mainland China and is conducted annually by the National Health Commission and carried out using a stratified, multistage, and probability proportion to size sampling (PPS) method. It targets individuals in the Chinese floating population aged 15 or above who have lived in the local area for over one year and are not registered there. The information in this survey includes basic demographic characteristics, employment, mobility and residence willingness, health status, and medical insurance enrolment. Therefore, we selected this authoritative survey as the data source for this research.

The raw dataset included 286716 observations. To obtain suitable data for our analysis, we first excluded respondents who only flowed within the province or crossed the national border (139455 were excluded). We then excluded the data if the number of respondents at the municipal level was smaller than 10 or the city was not in the sampling district in both 2017 and 2018 (10127 were excluded). In addition, the sample consisted of the respondents whose detailed demographic, socioeconomic, health status, and insurance information were available (3946 were excluded), and we retained only the respondents who enrolled in public medical insurance (11127 were excluded). After this selection process, we ultimately retained 122061 observations (62499 in 2017 and 59562 in 2018).

The hospital data were collected from the website of the Ministry of Human Resources and Social Security of China. Each month, this department announces the information of hospitals in each city that immediately reimburse trans-provincial inpatients for treatment, including the name and tier of the hospitals. The CMDS-2017 and CMDS-2018 were conducted in May, 2017 and May, 2018 respectively, thus we used the hospitals data announced on the corresponding date. Based on these data, we were able to calculate the number of qualified hospitals in each city and judge whether the city carried out this policy change by the date of the survey. The number of reserved cities is 262 per year, and 33.21% of them carried out this immediate reimbursement policy before May 2017 (see more details in Appendix Table 7). That is, not all the cities immediately realized it at the time when the central government released this policy change. But at the end of May 2018, all the cities in our study finished this target. We then matched the city-level hospital data with the individual-level data based on city and year variables.

### Measures

#### Dependent variables

We used self-rated health (SRH) to represent the health status of each respondent. In the CMDS, self-rated health is measured with the following question: “Would you say your health is…” with four options (good, basically good, poor but able to care for self, and poor and unable to care for self). Household income was then employed to measure socioeconomic status. To this end, the CDMS includes the following question: “In the past year, what was the average monthly gross income of your family?”.

Besides, we conducted a simple mechanism analysis. First, to indicate the inpatient utilization level of each individual, we defined a binary dummy variable that equals 1 if individual utilized inpatient in destination city and equals 0 otherwise, based on the following question:“Have you ever utilized inpatient care in the past year? If yes, where was the hospital that you visited?” Then, we calculated total inpatient expenditure and reimbursement for each individual based on the following question: “If you utilized inpatient services in the destination city, how much was the total spending and out-of-pocket payment?”.

#### Independent variables

The independent variable of interest for this study is the implementation of the policy change on immediate reimbursement. To better reflect the degree and intensity of this policy change, we used the number of qualified hospitals that can provide immediate reimbursement when the insured utilizes trans-provincial inpatient treatments to be the indicator. Besides, we also stratified total qualified hospitals into primary hospitals, secondary hospitals, and tertiary hospitals to examine whether the impact of the policy change was heterogeneous by hospital type.

Our analysis also controlled for a set of factors that may have influenced on health statuses. When we conducted the analysis at the individual level, sex, age, hukou type, marriage status, education level, migrating reason, and willingness to settle were used as covariates, which were considered potential confounders. Marriage status was categorized into three groups: unmarried, married, and others. Education level was categorized into four groups, including no formal education, elementary school, high school, and college or above. Migrating reason was categorized into four groups: work, business, family, and others. When conducting the analysis at the city level, we used the Gini coefficient, average age, male ratio, marriage rate, proportion of rural population, average years of schooling, and proportion of willingness to settle as covariates. Detailed definitions of all variables are presented in Table 1.

**Table 1:** Variable definition

Variables	Definitions
Self-rated health_it_ (SRH)	An ordinal variable that equals 3, 2, 1 or 0 if individual i rates its health condition at time t as good, basically good, poor but able to care for self, or poor and unable to care for self, respectively
Income_it_	The individual i’s family income at time t
Inpatient utilization_i_	A dummy variable that equals 1 if individual utilized inpatient care in destination city in 2018 and equals 0 otherwise
Total expenditure_i_	The individual i’s total inpatient expenditure in 2018
Total reimbursement_i_	The individual i’s total inpatient reimbursement in 2018
Qualified hospitals_jt_	The number of qualified hospitals in the city j that can provide immediate reimbursement when the insured utilizes trans-provincial inpatient treatments
Age_it_	The individual i’s age at time t
Gender_it_	A dummy variable that equals 1 if individual i is male, and equals 0 otherwise
Hukou type_it_	A dummy variable that equals 1 if individual i owns rural hukou, equals 0 otherwise
Marriage status_it_	The individual i’s marriage status at time t. It’s categorized into three groups: unmarried, married, and others
Education level_it_	The individual i’s education level at time t. It’s categorized into four groups, including no formal education, elementary school, high school, and college or above
Migrating reasons_it_	The individual i’s migrating reason at time t. It’s categorized into four groups: work, business, family, and others
Willingness to settle_it_	A dummy variable that equals 1 if individual i has willingness to settle, and equals 0 otherwise
Wagstaff index_jt_	The value of Wagstaff index in city j at time t
Gini coefficient_jt_	The value of gini coefficient in city j at time t
Average age_jt_	The average ages of whole floating population in city j at time t
Male ratio_jt_	The number of male in the whole floating population in city j at time t
Marriage rate_jt_	The number of married people in the whole floating population in city j at time t
Proportion of rural population_jt_	The number of people with rural hukou in total floating population in city j at time t
Years of schooling_jt_	The average years of schooling of whole floating population in city j at time t
Proportion of willingness to settle_jt_	The number of people who have the willingness to settle in total floating population in city j at time t

### Statistical analysis

As depicted above, only 87 in 262 cities conducted this policy change before May, 2017. The asymptotic executing processes of this policy change provided quasi-experimental research design to evaluate causal relationships in our study. Due to the fact that whether to implement the reform is decided by the local government and not directed determined by individuals and it’s non-existent that the reform will be canceled after implementation, we built up the framework to employ the multi-period difference-in-differences estimation strategy [47]. Besides, as mentioned above, we mainly used the number of qualified hospitals to reflect the degree and intensity of policy change. It’s also a generalized DID setting, which was consistent as the study of Qian [48].

We first used the individual-level pooled cross-sectional data and employed the following empirical strategy to investigate the relationship between the number of qualified hospitals and the health status of the trans-provincial floating population within a generalized and multi-period DID framework.

1 $${SRH}_{ijt}=\alpha +\beta {Treat}_{jt}+\gamma {Income}_{ijt}+{X}_{it}^{^{\prime}}\psi +{\varphi }_{j}+{\tau }_{t}+{\varepsilon }_{ijt}$$

where *SRH*_*ijt*_ denotes the health status of enrollee *i* who lives in the city j at time t. To this end, for SRH variable is ordinal, we used the ordered logistic model to estimate the odds ratio (OR) of the intervention, which can be interpreted as the effect of treatment on the odds ratio of health status. *Treat*_*jt*_ represents the number of qualified hospitals in the city j at time t. If the cities that have not implemented the policy change, *Treat*_*jt*_ will take a value of 0. *β* measures the impact of this policy change on SRH. *X* is a vector of control variables. ${\varphi }_{j}$ and *τ*_*t*_ represent the region and calendar-year fixed effects. After adding these variables, the basic generalized and multi-period difference-in-differences is set up in our study. ${\varepsilon }_{ijt}$ is the error term.

Then to analyze whether there is a moderating effect of the number of qualified hospitals on the relationship between income level and health status of individuals, we added the cross term of the number of qualified hospitals and income level and the regression model transferred into the following form:

2 $${SRH}_{ijt}=\alpha +\beta {Treat}_{jt}+\gamma {Income}_{ijt}+\rho {Treat}_{jt}\times {Income}_{ijt}+{X}_{it}^{^{\prime}}\psi +{\varphi }_{j}+{\tau }_{t}+{\varepsilon }_{ijt}$$

Here, if $\beta$ and $\gamma$ were significantly positive while $\rho$ was significantly negative, it could be implied that this policy change helped reduce the socioeconomic inequality in health among the floating population.

Besides, because the floating population usually lives within the range of the city, we then calculated the index of socioeconomic inequality in health at the city level, transformed the individual-level data into city-level balanced panel data and examined the policy effects. Here, we used Wagstaff index (WI) to directly measure socioeconomic inequality in health to address the problem that the health variable is not measured on the same scale as income [16]. The Wagstaff index is defined as follows:

3 $$WI=\frac{2}{n\overline{Y} }\sum\nolimits_{i=1}^{n}{Y}_{i}{R}_{i}-1$$

where ${Y}_{i}$ represents individual $i$'s health condition and $\overline{Y }$ is its mean value in the population. ${R}_{i}$ is the fractional rank of the $i$ th individual in the income distribution, $n$ denotes the sample size. WI ranges from − 1 to 1. In this study, we used positive health variables; thus, a positive or negative WI indicates that good health is more concentrated among those with higher or lower socioeconomic rank, respectively, which consequently indicates pro-rich or pro-poor health inequality, respectively.

Based on these settings, we examined the effects of policy change on WI within the same framework.

4 $${WI}_{jt}=\alpha +\beta {Treat}_{jt}+{X}_{jt}^{^{\prime}}\psi +{\varphi }_{j}+{\tau }_{t}+{\varepsilon }_{ijt}$$

where WI_jt_ denotes the level of health equality in the city j at time t. Other specifications are the same as above.

In addition, we conducted several robustness checks. First, the key assumption in our DID specification is that the selection of pilot cities is uncorrelated with other determinants of changes in health outcomes, which is also known as the parallel trend assumption. Due to the fact that only two time periods were used and multi-period difference-in-differences method was conducted in our study, we tested this assumption by the following method: by only using the data in 2018, we compared the SRH between the individuals who lived in the cities that implemented the immediate reimbursement before May, 2017 and the individuals who lived in the cities that implemented this policy change after May, 2017. Second, we defined a binary dummy to represent this policy change. That is, it equals 1 if the city implemented the immediate reimbursement, and equals 0 otherwise. Besides, considering that there are stronger assumption to ensure the validity of nonlinear model, we replaced the nonlinear ordered logistic model with the generalized linear model with fixed effects. Finally, to strengthen causal interpretation, we also adapt the propensity score matching (PSM) method to pick up comparable groups. Seven covariates were selected based on the data availability, including per capita GDP, whether there was net annual inflow, whether there was a provincial capital city or province-level municipality, whether there was any other experimental city in the same province, and the proportions of the number of experimental cities in total cities in the same province, number of hospitals per 1,000 persons and population density.

Finally, we clustered the standard errors at the individual or district level to correct for possible autocorrelation and heteroscedasticity. All the variables that involve price have been deflated by local Medical Consumer Price Index to eliminate the impact of price.

## Results

### Descriptive statistics

Tables 2 and 3 show the descriptive statistics of main variables used in this study. At the individual level (Table 1), among those 122061 respondents, most of them thought their health statuses were good for mean SRH was 3.838. Average income level was 792.5 per month. Average age was 37.3. 53.4% were male. 84.2% had urban hukou. 13.2% were unmarried. 83.1% were married. 15.5%, 46% and 19.9% only received primary, junior high and high school education respectively. 16% owned bachelor’s degree or above. 61.9% migrated for work. 9.7% migrated for family. 82.5% had the willingness to settle. In addition, at the city level (Table 2), the average number of qualified hospitals was 18.4. Among them, 23.9% were primary hospitals. 27.2% were tertiary hospitals. The average value of Wagstaff index was 0.144 (*P*<0.01), indicating pro-rich health inequality existed in most cities.

**Table 2:** Descriptive statistics at the individual level

	Mean	SD	Min	Max
SRH	3.838	0.420	1.000	4.000
Income	792.5	541.8	108.0	30,000
Ever utilizing inpatient services	0.018	0.134	0	1
Total expenditure	355.4	4235.4	0	250,000
Total reimbursement	130.3	1918.0	0	150,000
Age	37.3	11.0	15.0	90.0
Gender (Male = 1)	0.534	0.499	0.000	1.000
Hukou type (Rural = 1)	0.842	0.365	0.000	1.000
Marriage (Unmarried = 1)	0.132	0.338	0.000	1.000
Marriage (Married = 1)	0.831	0.375	0.000	1.000
Education (Primary school = 1)	0.155	0.362	0.000	1.000
Education (Junior high school = 1)	0.460	0.498	0.000	1.000
Education (High school = 1)	0.199	0.399	0.000	1.000
Education (College or above = 1)	0.160	0.367	0.000	1.000
Migrating reason (Work = 1)	0.619	0.486	0.000	1.000
Migrating reason (Business = 1)	0.250	0.433	0.000	1.000
Migrating reason (Family = 1)	0.097	0.297	0.000	1.000
Willingness to settle (Yes = 1)	0.825	0.380	0.000	1.000

**Table 3:** Descriptive statistics at the city level

	Mean (Composition ratio %)	SD	Min	Max
The number of qualified hospitals	18.4	43.8	0.0	673.0
Primary hospitals	4.4 (23.9)	26.4	0.0	440.0
Secondary hospitals	9.1 (48.9)	13.7	0.0	153.0
Tertiary hospitals	5.0 (27.2)	9.5	0.0	106.0
Wagstaff index	0.144	0.293	-0.928	0.972
Gini coefficient	0.279	0.055	0.109	0.448
Average age (years old)	38.2	3.1	28.3	55.8
Male ratio	0.544	0.083	0.222	0.905
Marriage rate	0.860	0.087	0.256	1.000
Proportion of rural population	0.867	0.109	0.206	1.000
Years of schooling (years)	9.603	1.046	5.625	14.971
Proportion of willingness to settle	0.798	0.102	0.209	1.000

We further conducted a T test to examine whether the mean value of Wagstaff index was significantly greater than 0. The results showed that T value was -11.29 and its P value was 0

### Main results

Table 4 reports the regression results obtained using the ordered logistic model and individual-level pooled cross-sectional data. The odds ratio (OR) of the number of qualified hospitals was 1.043 (95%CI, 1.017-1.069, *P*=0.001), indicating that the health status of the floating population significantly improved as the number of qualified hospitals increased. In addition, the better health status associated with a higher income level is unsurprising, which further indicates distinct pro-rich inequality in health in China and that the OR of income was 1.280 (95% CI, 1.221–1.342, *P* < 0.001). Finally, the OR of the cross term of the number of qualified hospitals and income was 0.955 (95% CI, 0.940–0.971, *P* < 0.001); that is, substitution effects were present, and the lower the income, the better the number of qualified hospitals' effect on health improvement. We also stratified total qualified hospitals into primary hospitals, secondary hospitals, and tertiary hospitals and replaced the number of total hospitals with the number of primary hospitals, secondary hospitals, and tertiary hospitals in the model. The results show that only an increase in the number of qualified tertiary hospitals could help promote the health of the floating population. The OR was 2.211 (95% CI, 1.528-3.201, *P* < 0.001). Similarly, the lower the income, the better the number of qualified tertiary hospitals' effect on health improvement, and the OR was 0.641 (95% CI, 0.485–0.847, *P* = 0.002).

**Table 4:** The moderating effects of the number of qualified hospitals on the relationship between income and health (*N*=122061)

	OR (95% CI)	*P* value	OR (95% CI)	*P* value
Total hospitals	1.043 (1.017–1.069)	0.001	-	-
Primary hospitals	-	-	0.899 (0.812–0.994)	0.038
Secondary hospitals	-	-	1.094 (0829–1.443)	0.527
Tertiary hospitals	-	-	2.211 (1.528–3.201)	0.000
Income	1.280 (1.221–1.342)	0.000	1.332 (1.257–1.411)	0.000
Hospital × Income	0.955 (0.940–0.971)	0.000	-	-
Primary hospitals × Income	-	-	1.043 (0.753–1.150)	0.366
Secondary hospitals × Income	-	-	0.930 (0.753–1.150)	0.504
Tertiary hospitals × Income	-	-	0.641 (0.485–0.847)	0.002
Age	0.941 (0.939–0.943)	0.000	0.941 (0.939–0.943)	0.000
Gender (Male = 1)	1.219 (1.175–1.265)	0.000	1.219 (1.1747–1.264)	0.000
Hukou type (Rural = 1)	1.065 (1.008–1.126)	0.026	1.065 (1.007–1.126)	0.027
Marriage (Unmarried = 1)	1.179 (1.060–1.312)	0.002	1.180 (1.060–1.312)	0.002
Marriage (Married = 1)	1.166 (1.074–1.265)	0.000	1.167 (1.075–1.266)	0.000
Education (Primary school = 1)	1.299 (1.184–1.424)	0.000	1.296 (1.182–1.422)	0.000
Education (Junior high school = 1)	1.695 (1.548–1.857)	0.000	1.693 (1.545–1.854)	0.000
Education (High school = 1)	1.750 (1.585–1.932)	0.000	1.749 (1.584–1.930)	0.000
Education (College or above = 1)	1.772 (1.586–1.979)	0.000	1.773 (1.587–1.981)	0.000
Migrating reasons (Work = 1)	1.474 (1.348–1.612)	0.000	1.473 (1.347–1.610)	0.000
Migrating reasons (Business = 1)	1.792 (1.629–1.970)	0.000	1.789 (1.627–1.967)	0.000
Migrating reasons (Family = 1)	0.974 (0.884–1.073)	0.595	0.972 (0.883–1.071)	0.571
Willingness to settle (Yes = 1)	1.168 (1.117–1.221)	0.000	1.167 (1.117–1.220)	0.000
Year fixed effects	control		control	
District fixed effects	control		control	

The dependent variable is SRH. Here, we used individual-level cross-sectional data. The ordered logistic model was employed, and data were also adjusted for year and district effects. The standard errors were clustered at the individual level. The 95% confident level were reported in parentheses

We then used city-level balanced panel data and employed a generalized linear model to directly estimate the impact of the number of qualified hospitals on the WI of health. The corresponding regression results are reported in Table 5, which show that the coefficient of the number of qualified hospitals was -0.035 (95% CI, -0.074–0.005, *P* = 0.083), indicating a negative relationship between the number of qualified hospitals and socioeconomic inequality in health. Moreover, socioeconomic inequality in health also significantly decreased as the number of qualified tertiary hospitals increased, and the coefficient was -0.316 (CI, -0.617–0.015, *P* = 0.040). The impact of primary and secondary hospitals was also not significant.

**Table 5:** The relationship between the number of qualified hospitals and socioeconomic inequality in health (*N*=524)

	Coefficient (95% CI)	*P* value	Coefficient (95% CI)	*P* value
Hospitals	-0.035 (-0.074–0.005)	0.083	-	
Primary hospitals	-		-0.007 (-0.093–0.077)	0.856
Secondary hospitals	-		0.089 (-0.204–0.382)	0.542
Tertiary hospitals	-		-0.316 (-0.617—-0.015)	0.040
Gini coefficient	0.184 (-0.3906–0.7580)	0.519	0.2138 (-0.3564–0.7840)	0.450
Average age	0.004 (-0.007–0.015)	0.457	0.003 (-0.008–0.015)	0.542
Male ratio	-0.026 (-0.379–0.327)	0.881	-0.030 (-0.384–0.323)	0.863
Marriage rate	-0.022 (-0.456–0.412)	0.919	-0.030 (-0.460–0.399)	0.887
Proportion of rural population	-0.210 (-0.638–0.218)	0.325	-0.208 (-0.641–0.225)	0.336
Years of schooling	0.005 (-0.035–0.045)	0.789	0.008 (-0.033–0.048)	0.697
Proportion of willingness to settle	-0.329 (-0.656—-0.001)	0.050	-0.320 (-0.645–0.006)	0.054
Year fixed effects	control		control	
District fixed effects	control		control	

The dependent variable is WI. Here, we used city-level balanced panel data. The generalized linear model was employed. The data were also adjusted for year and district effects. The standard errors were clustered at the district level. The 95% confident level were reported in parentheses

### Mechanism analysis

We further explored the mechanism underlying this influence, and the results are given in Table 6. Only CMDS-2018 provided information on inpatient utilization and reimbursement; therefore, we used cross-sectional data herein. By regressing these data on the number of qualified hospitals and other covariates, we found that on the one hand, although the number of total qualified hospitals did not make effects, the lower the income, the better the number of qualified tertiary hospitals' effect on inpatient utilization (*P* < 0.1). On the other hand, total inpatient expenditure and reimbursement significantly improved as the number of qualified hospitals increased, and this relationship was more effective in the relatively low-income group, as the coefficients of the cross term were -15.7 (95% CI, -23.9-7.5, *P* < 0.001) and -5.7 (95% CI, -11.3–0.1, *P* < 0.05), respectively. Similar results were found when using the number of tertiary hospitals as the core independent variable, and the coefficients of the cross term were -139.5 (95%CI, -187.7–91.4, *P* < 0.001) and -52.3 (95% CI, -79.0–25.6, *P* < 0.001), respectively.

**Table 6:** Mechanism analysis

	Coefficient (95% CI)	*P* value	Coefficient (95% CI)	*P* value
A:Whether utilizing inpatient services in the destination city (*N* = 59,562)				
Total hospitals	-0.002 (-0.003–0.001)	0.544	-	-
Primary hospitals	-	-	-0.002 (-0.005–0.001)	0.137
Secondary hospitals	-	-	0.001 (-0.005–0.008)	0.680
Tertiary hospitals	-	-	-0.005 (-0.013–0.003)	0.214
Income	0.016 (-0.164–0.195)	0.545	-0.047 (-0.263– 0.169)	0.669
Hospital × Income	0.0003 (-0.0002–0.001)	0.257		-
Tertiary hospitals × Income	-	-	0.003 (0.000–0.007)	0.045
B:Total inpatient expenditure (*N* = 1083)				
Total hospitals	34.6 (25.1–44.0)	0.000	-	-
Primary hospitals	-	-	25.6 (-2.6–53.8)	0.075
Secondary hospitals	-	-	-33.0 (-103.5–37.5)	0.356
Tertiary hospitals	-	-	217.4 (105.4–329.4)	0.000
Income	4853.5 (1039.6–8667.4)	0.000	6491.8 (2507.7–10,476.1)	0.002
Hospital × Income	-15.7 (-23.9–7.5)	0.000	-	-
Tertiary hospitals × Income	-	-	-139.5 (-187.7–91.4)	0.000
C:Total inpatient reimbursement (*N* = 1083)				
Total hospitals	16.7 (7.8–25.6)	0.000	-	-
Primary hospitals	-	-	17.3 (5.5–29.2)	0.004
Secondary hospitals	-	-	-11.8 (-34.6–10.9)	0.306
Tertiary hospitals	-	-	69.9 (28.7–111.1)	0.001
Income	1220.5 (-41.8–2482.8)	0.058	1879.3 (590.3–3168.3)	0.005
Hospital × Income	-5.7 (-11.3–0.1)	0.047	-	-
Tertiary hospitals × Income	-	-	-52.3 (-79.0–25.6)	0.000

First, in Panel A, we examined the relationship between the number of qualified hospitals and inpatient utilization. Dependent variable is a binary dummy variable that equals 1 if individual utilized inpatient in destination city and equals 0 otherwise. Then, we analyzed the relationship between the number of qualified hospitals and inpatient expenditure within the group who has ever utilized inpatient services in the destination city. 1083 individuals were kept. Dependent variable is total expenditure and reimbursement in Panel B and Panel C, respectively. All other specifications are the same as those in Table 4

### Robustness checks

To ensure the solidity of our results, we conducted the robustness checks form the following three aspects. First, we tested parallel trend assumption as depicted above and obtained the results shown in Panel A of Appendix Table 8. As we can see, in 2018 when all cities realized immediate reimbursement of medical insurance, the health statuses of floating populations showed no differences, that is, the pilot cities were uncorrelated with the health outcomes of the individuals who lived here and the parallel trend assumption was met in our study. Second, we used a binary dummy to represent this policy change. Within this setting, we obtained the results reported in Panel B. Besides, we employed the generalized linear model with fixed effects, and corresponding results were shown in Panel C. Finally, we adapt the propensity score matching (PSM) method to pick up comparable groups. By using kernel matching and radius matching respectively, we obtained the results shown in Panel C and Panel D. The results were consistent with those in our main analysis. Therefore, our results were robust in general.

## Discussion and conclusion

The health status and health inequality of the floating population have garnered considerable concern around the world. Improving their medical accessibility has proved to be an effective method and became a prominent goal on the development of public health policy worldwide. China implemented immediate medical insurance reimbursement for trans-provincial inpatient treatments in 2017. Under a quasi-experimental research design, this study was one of the first to attempt to examine the effects of this policy change on socioeconomic inequality in health among the floating population. By using two waves of data from the CMDS collected in 2017 and 2018 as well as the administrative data of hospitals and employing the generalized and multi-period difference-in-differences estimation strategy, we identified significant pro-rich inequality in health among the floating population in China. However, this policy change made utilizing inpatient services and obtaining reimbursement from medical insurance more convenient for this population. As the number of hospitals that could provide immediate reimbursement increased, the health status of the floating population significantly improved. In addition, the lower the income, the better the number of qualified hospitals' effect on health improvement; that is, it also helped to reduce socioeconomic inequality in health. These effects were more effective in tertiary care than other hospitals. As the number of qualified tertiary hospitals increased, the health status of the floating population considerably improved, and the health disparities further decreased. The effects were insignificant when the number of qualified primary and secondary hospitals increased. Finally, the mechanistic analysis further indicated that this policy change improved inpatient utilization and reduced the medical burdens of the floating population meanwhile. These impacts were greater in the relatively low-income group and promoted socioeconomic equality in health.

First, our finding that there was significant pro-rich health inequality among the floating population is consistent with prior studies (e.g. [49, 50]). First, high earners usually receive better health education and develop better habits which bring them health advantages. Carnazza et al. [49] investigated the association between income and the habit of smoking in 30 European countries, and found that smoking is a habit which is mainly rooted in the lowest part of the income distribution both at individual and country level. Second, environmental pollution will also enlarge socioeconomic health inequality. Liao et al. [51] found in the face of environmental pollution, the rich pay more attention to their health condition and increase beneficial health behaviors, such as increasing sleeping hours, compared with the poor. Finally, the rich usually enjoy better medical accessibility as well as health security meanwhile [15, 52]. Shang and Wei [50] showed significant socioeconomic differences in the areas of self-rated health, functional limitations, and reported chronic diseases in China. Besides, socioeconomic conditions such as income level will determine the level of health security that can be obtained. Tan et al. [53] found that UEBMI, which offers the most generous benefits, incurs the highest total costs prior to reimbursement when compared to other schemes. For floating population in China, they have to face unstable jobs and incomes, broken social relationships, changed lifestyle and living environment, as well as limited health security, which bring them greater health risks. However, as our results indicate, there still existed significant pro-rich health inequality, that is, floating populations with higher income level still have health advantages, and above mechanisms remain true in this vulnerable group.

Second, our findings are important given the ongoing debates about the impacts of medical insurance on healthcare equality. Most studies found medical insurance help promote health care utilization and take greater effects in the groups with lower socio-economic situations (e.g. [21, 41]). In China, the problems caused by fragmentation of medical insurance schemes already received the attention of policy-makers, it advanced the integration of rural and urban resident medical insurance scheme in 2016. Emerging studies examine the impacts of this policy change. Ren et al. [41] suggested that URRBMI reform in China makes contribution to the pro-poor inequity in the outpatient benefit rate. However, some studies found the opposite results. For example, Yang et al. [51] demonstrated that the inequality in utilization remained unchanged after the policy change because of urban–rural inequality in access to health care. That is, the level of equality in health care will affect the intervention effects from medical insurance. In fact, in China, except for the uneven distribution of medical resources, there are definite categories that specify the basic drugs, clinical items and medical service facilities that can be reimbursed. Contingent on economic development and financing capacity, benefit packages and categories vary considerably across cities. After immediate reimbursement was realized, the floating population could enjoy the reimbursement ratios of the place where they got enrolled, while had to meet the categories of the place where they utilized medical services [30]. Any services out of the categories could not get reimbursed, which might even cause the actual reimbursement ratios to be much lower than the level stipulated by the policy and effects of this policy change become indefinite. However, even with the aforementioned limitations, we found this policy change still have positive impacts on the health of floating population and play a greater role in the group with lower income level. The reasons may lie in the fact that this vulnerable group usually underuses medical services and its price elasticity of demand for it is negative and relatively greater in absolute value. After immediate reimbursement was realized, the waiting time for reimbursement was considerably shortened [33], and medical burdens for floating populations were ameliorated. Under these circumstances, this population was able to access more medical care in a timely manner, which helped to improve health. Besides, the worse the socioeconomic conditions were, the more responsive they were to the price change. Thus, the health inequality would decrease after the policy change.

Finally, our finding that increased primary care accessibility did not impact the health status of the floating population contrasts those of previous research. For example, Purdy et al. [42] proposed that improving the accessibility of primary care can reduce unnecessary hospitalization, so relevant policy interventions such as telemedicine should be adopted in NHS. Trivedi et al. [43] found that raising cost sharing for ambulatory care among elderly patients in Medicare plans may have adverse health consequences and may increase total spending on health care. We found it not helping may be due to fact that the immediate reimbursement policy that launched in 2017 was restricted to inpatient treatment. In China, patients usually prefer to visit larger, more specialized hospitals because they perceive these hospitals as offering higher-quality treatments [54]. When patients are in poor health and must utilize inpatient care, this medical behaviour could be more common and pronounced.

This paper is beneficial for relative policy formulations in several ways. First, our results indicated that the health status of the floating population improved and health inequality was reduced as reimbursement accessibility improved; thus, further promoting and perfecting this policy is worthwhile. For example, the cost-sharing ratios should be set more reasonably. Some cities still increase the cost-sharing ratios when patients utilize medical services outside the registration place to control costs even though reimbursement is immediate. However, the floating population is usually subject to poor and unstable economic conditions, and this setting may prohibit them from utilizing medical services and cause poor health outcomes. In addition, we found that increases in the number of qualified primary hospitals had no impact, primarily because outpatient care still could not be reimbursed. However, as ageing accelerates in the population, more and more elderly are leaving their hometowns and living with their children in other cities. In fact, most of this population suffers from chronic diseases, such as hypertension and diabetes, and they consequently require more primary care. Therefore, this policy should cover outpatient spending as an increasing number of cities do in China. Finally, the number of qualified hospitals continues to increase, but the government should enhance the management and assessment of the hospitals in service quality, cost control, etc. to ensure that the floating population can obtain equal and accessible medical services and to maintain the sustainability of medical insurance funds.

Our study still has some limitations. We failed to use more data before the policy change for CMDS-2016, which does not provide the health information of participants, and we cannot consider any psychological factors except for SRH. In addition, limited by the dataset, we tried to analyze the underlying mechanisms, but it is still not rigorous to complete accurate causal inferences. Finally, we only targeted the insured floating population and did not consider the differences of reimbursement ratios among cities. Therefore, whether this policy change will increase the willingness to enrol in the medical insurance scheme, and how the reimbursement ratios impact the health outcomes of the floating population require further study.

## Data Availability

The authors do not have permission to share data.

## Appendix

**Table 7:** The implementation process of immediate reimbursement for cities in our sample

	Cities
The cities that realized the immediate reimbursement in May, 2017 or before (87)	Aksu, Altay, Baicheng, Baishan, Bayingolin, Beijing, Beihai, Benxi, Bortala, Cangzhou, Changchun, Changji, Changsha, Changzhou, Chengde, Chengdu, Chongqing, Dalian, Daxinganling, Dezhou, Dongying, Fuzhou, Guangzhou, Guiyang, Guyuan, Hainan(P), Hangzhou, Harbin, Heifei, Huludao, Ili, Jilin, Jinan, Jining, Karamay, Kashgar, Kizilsu, Kumul, Kunming, Langfang, Lianyungang, Ma’anshan, Nanjing, Nanning, Nantong, Neijiang, Panjin, Panzhihua, Qiannan, Qingdao, Qinhuangdao, Shenyang, Shijiazhuang, Shizuishan, Siping, Songyuan, Suqian, Suzhou, Taiyuan, Taizhou, Tangshan, Tarbagatay, Tianjin, Tongchuan, Turpan, Urumqi, Xianning, Xianyang, Xi’an, Xining, Xuzhou, Weifang, Weihai, Wuhan, Wuxi, Wuzhong, Wuzhou, Yan’an, Yanbian, Yantai, Yangzhou, Yinchuan, Yuxi, Zhengzhou, Zhenjiang, Zhongwei, Zunyi
The cities that realized the immediate reimbursement after May, 2017 and before May, 2018 (175)	Alxa, Ankang, Anqing, Anshan, Anshun, Anyang, Baise, Baiyin, Baoding, Baoji, Baoshan, Baotou, Bayannur, Bengbu, Bijie, Changde, Changzhi, Chaozhou, Chenzhou, Chifeng, Chuxiong, Dali, Dandong, Daqing, Datong, Dehong, Deyang, Dingxi, Dongguan, Enshi, Ezhou, Foshan, Fuyang, Fuzhou, Gannan, Ganzhou, Guang’an, Guilin, Guoluo, Haibei, Haixi, Handan, Hanzhong, Hechi, Heihe, Hengshui, Hengyang, Heyuan, Hinggan, Huzhou, Hohhot, Holonbuyr, Honghe, Huaihua, Huanggang, Huangnan, Huangshi, Huizhou, Jiamusi, Jiangmen, Jiaozuo, Jiaxing, Jiayuguan, Jieyang, Jincheng, Jingdezhen, Jinhua, Jingzhou, Jinzhong, Jinzhou, Jiujiang, Jixi, Kaifeng, Lanzhou, Laibin, Liangshan, Liaoyang, Lijiang, Lincang, Linfen, Linyi, Lishui, Liu’an, Liupanshui, Liuzhou, Longyan, Luohe, Luoyang, Maoming, Meishan, Mianyang, Mudanjiang, Nanchang, Nanchong, Nanping, Nanyang, Ningbo, Ningde, Nujiang, Ordos, Pingdingshan, Pingliang, Pingxiang, Pu’er, Putian, Puyang, Qingyang, Qiandongnan, Qiangxinan, Qinzhou, Qitaihe, Quanzhou, Qujing, Quzhou, Sanming, Shanghai, Shangluo, Shangqiu, Shangrao, Shantou, Shanwei, Shaoguan, Shaoxing, Shenzhen, Shiyan, Shuangyashan, Shuozhou, Suizhou, Qingyuan, Taizhou, Tianshui, Tibet, Tieling, Tongliao, Tongling, Tongren, Ulanqab, Weinan, Wenshan, Wenzhou, Wuhai, Wuhu, Xiamen, Xiantao, Xiangtan, Xiangxi, Xiangyang, Xiaogan, Xilingol, Xinxiang, Xinzhou, Xishuangbanna, Xuancheng, Yangjiang, Yangquan, Yibin, Yichang, Yichun, Yingkou, Yongzhou, Yueyang, Yulin(S), Yulin(G), Yuncheng, Yunfu, Yushu, Zhangjiakou, Zhangzhou, Zhaoqing, Zhaotong, Zhongshan, Zhoushan, Zhuhai, Zhumadian, Zhuzhou

**Table 8:** Robustness checks

	A: Parallel trend test (*N* = 59,562)		B: Multi-period DID (*N* = 122,061)		C: Generalized linear model (*N* = 122,061)		D: PSM-DID with kernel matching (*N* = 102,427)		E: PSM-DID with radius matching (*N* = 102,963)	
	OR (95% CI)	*P* value	OR (95% CI)	*P* value	OR (95% CI)	*P* value	OR (95% CI)	*P* value	OR (95% CI)	*P* value
Policy change	0.263 (0.050–1.370)	0.113	1.1149 (1.013–1.227)	0.026	0.004 (0.001–0.007)	0.004	1.036 (1.010–1.062)	0.007	1.037 (1.010–1.063)	0.006
Income	1.120 (1.030–1.218)	0.008	1.395 (1.286–1.512)	0.000	0.033 (0.028–0.038)	0.000	1.256 (1.195–1.321)	0.000	1.257 (1.196–1.322)	0.000
Policy change × Income	1.060 (0.936–1.200)	0.360	0.829 (0.758–0.907)	0.000	-0.006 (-0.008–0.004)	0.000	0.959 (0.943–0.975)	0.000	0.959 (0.943–0.975)	0.000
Age	0.940 (0.937–0.942)	0.000	0.941 (0.939–0.943)	0.000	-0.010 (-0.010–0.010)	0.000	0.941 (0.939–0.943)	0.000	0.941 (0.939–0.943)	0.000
Gender (Male = 1)	1.208 (1.142–1.278)	0.000	1.220 (1.175–1.265)	0.000	0.027 (0.022–0.032)	0.000	1.197 (1.150–1.246)	0.000	1.198 (1.151–1.246)	0.000
Hukou type (Rural = 1)	1.048 (0.965–1.138)	0.268	1.069 (1.011–1.129)	0.019	0.008 (0.001–0.015)	0.029	1.066 (1.004–1.131)	0.036	1.064 (1.002–1.129)	0.042
Marriage (Unmarried = 1)	1.143 (0.974–1.342)	0.102	1.181 (1.062–1.314)	0.002	0.011 (-0.003–0.026)	0.131	1.152 (1.026–1.292)	0.016	1.147 (1.022–1.286)	0.020
Marriage (Married = 1)	1.210 (1.073–1.366)	0.002	1.170 (1.078–1.269)	0.000	0.052 (0.037–0.066)	0.000	1.144 (1.046–1.250)	0.003	1.141 (1.044–1.246)	0.004
Education (Primary school = 1)	1.343 (1.166–1.546)	0.000	1.300 (1.185–1.426)	0.000	0.105 (0.081–0.130)	0.000	1.280 (1.155–1.418)	0.000	1.281 (1.156–1.419)	0.000
Education (Junior high school = 1)	1.689 (1.470–1.940)	0.000	1.700 (1.552–1.861)	0.000	0.158 (0.135–0.182)	0.000	1.689 (1.527–1.869)	0.000	1.688 (1.526–1.868)	0.000
Education (High school = 1)	1.813 (1.560–2.108)	0.000	1.757 (1.591–1.939)	0.000	0.158 (0.135–0.182)	0.000	1.723 (1.544–1.922)	0.000	1.722 (1.543–1.921)	0.000
Education (College or above = 1)	1.842 (1.557–2.179)	0.000	1.769 (1.584–1.976)	0.000	0.159 (0.135–0.183)	0.000	1.743 (1.544–1.968)	0.000	1.729 (1.531–1.953)	0.000
Migrating reasons (Work = 1)	1.649 (1.449–1.877)	0.000	1.475 (1.349–1.613)	0.000	0.090 (0.075–0.106)	0.000	1.465 (1.331–1.613)	0.000	1.466 (1.331–1.613)	0.000
Migrating reasons (Business = 1)	1.981 (1.725–2.275)	0.000	1.797 (1.635–1.976)	0.000	0.118 (0.102–0.135)	0.000	1.790 (1.616–1.983)	0.000	1.793 (1.619–1.987)	0.000
Migrating reasons (Family = 1)	0.976 (0.848–1.123)	0.734	0.975 (0.885–1.074)	0.608	-0.009 (-0.027–0.009)	0.350	0.932 (0.840–1.034)	0.186	0.932 (0.840–1.034)	0.186
Willingness to settle (Yes = 1)	0.906 (0.873–0.940)	0.000	1.169 (1.118–1.221)	0.000	0.019 (0.013–0.026)	0.000	1.183 (1.128–1.242)	0.000	1.183 (1.128–1.242)	0.000
Year fixed effects	control		control		control		control		control	
District fixed effects	control		control		control		control		control	

In Panel A, we only used the data in 2018. In Panel B, we measured the policy change as a binary variable taking a value of 1 if the city implemented the policy change, and a value of 0 otherwise. In Panel C, we employed the generalized linear model. In Panel D and Panel E, we conducted the PSM-DID method. In Panel C, we used a with replacement kernel matching at the bandwidth of 0.06, and the kernel type is epan kernel. After matching, we obtained 50106 individuals from 50 cities which implemented the policy change before May 2017 and 52321 individuals from 163 cities which implemented the policy change after May 2017. In Panel D, we used a with replacement radius matching at the caliper value of 0.1. After matching, we obtained 50642 individuals from 52 cities which implemented the policy change before May 2017 and 52321 individuals from 163 cities which implemented the policy change after May 2017. Except those, other specifications are the same as that in Table 2. The 95% CI are shown in brackets. *** *p* < .01. ** *p* < .05. * *p* < .10
